# Sex as a Biological Factor in the Changes in Disease Patients During Ramadan Intermittent Fasting: A Systematic Review

**DOI:** 10.3389/fnut.2022.908674

**Published:** 2022-07-01

**Authors:** Rachida Roky, Nadia Aadil, Al Mehdi Krami, Brahim Benaji, Ikram Errabih, Dana N. Abdelrahim, MoezAlIslam Ezzat Faris

**Affiliations:** ^1^Department of Biology, Laboratory of Physiopathology, Molecular Genetics & Biotechnology, Faculty of Sciences Ain Chock, Health and Biotechnology Research Centre, Hassan II University of Casablanca, Casablanca, Morocco; ^2^Department of Biology, Laboratory of Microbiology, Pharmacology, Toxicology, Biotechnology and Environment Faculty of Sciences Ain Chock, Hassan II University of Casablanca, Casablanca, Morocco; ^3^Health Technologies Engineering Department, Research Group in Biomedical Engineering and Pharmaceutical Sciences, ENSAM, Mohammed V University, Agdal, Morocco; ^4^Gastroenterology Department, Ibn Sina Hospital, Mohammed V University, Agdal, Morocco; ^5^Department of Clinical Nutrition and Dietetics, Faculty of Pharmacy, Applied Science Private University, Amman, Jordan; ^6^Department of Clinical Nutrition and Dietetics, College of Health Sciences, Research Institute of Medical and Health Sciences (RIMHS), University of Sharjah, Sharjah, United Arab Emirates; ^7^Research Institute of Medical and Health Sciences (RIMHS), University of Sharjah, Sharjah, United Arab Emirates

**Keywords:** diabetes, renal, cardiovascular, gastrointestinal, FAST, gender, Islam

## Abstract

**Background:**

During Ramadan, many patients with diabetes, renal, cardiovascular, gastrointestinal diseases, headaches, and epilepsy choose to fast even against their doctor's advice. The impact of this intermittent fasting on health and disease could be different in men and women. The aim of this study was to determine the effect of sex as a factor in diseases outcomes of patients who opt to fast during Ramadan.

**Main Body:**

The articles included in this study reported data on six diseases: diabetes, renal, cardiovascular, gastrointestinal diseases, headaches, and epilepsy. A systematic search was performed on PubMed and Scopus for observational and clinical studies mentioning Ramadan, diabetes, renal, cardiovascular, gastrointestinal diseases, headaches, and epilepsy in both men and women. Data was extracted by two independent reviewers using a standardized data-collection form. From 381 original articles, 38 studies were selected, including 25,023 patients of which 44.4% were women. Sex-based differences were reported by 18 studies for several variables such as body mass index, blood glucose, the frequency of hypoglycemia, renal colic, mortality, thrombosis, and gastrointestinal diseases in patients fasting during Ramadan. Most of the differences between men and women were reported both in the baseline period before Ramadan and during Ramadan. Indeed, during the period outside Ramadan, the frequency of renal colic, cardiovascular, gastrointestinal diseases, were higher in men; while body mass index, Thrombosis, and headache were higher in women. In the remaining 21 studies, it was reported that the sex factor was not associated with the effect of Ramadan fasting in the frequency and other outcomes of these diseases.

**Conclusion:**

Currently, small attention is paid to sex as a determinant factor in patients while fasting during Ramadan. There appeared to be differences in the frequency and incidence of diseases in men and women during Ramadan. Closer attention to sex differences regarding the frequency and the progression of the diseases during fasting may help to improve patient care, especially to benefit those patients willing to fast during Ramadan.

## Introduction

Sex represents an important factor in biomedical science and may influence biological parameters, state of health, knowledge, attitude, and behavior in healthy and unhealthy participants. Consequently, rigorous science must include males and females (1). Sex and gender analysis promote rigorous and reproducible and responsible science (2). Several countries (e.g., USA, Canada, and the European Union) implemented policies in relation to sex as a biological variable in human research. For example, the US National Institute of Health implemented in 2016 a policy that expects scientists to account for the possible role of sex factors in human studies (3). In addition, Canada implemented a Sex and Gender-Based Analysis policy to ensure that health research in Canada addresses biological (sex) and sociocultural (gender) differences between diverse groups of people. Five years after the implementation of these policies, most of the studies have included women as participants in their research (3). Still, most of these studies (72.0%) did not mention whether sex was included in their analysis, did not report any sex-specific outcomes, and did not explain for not doing so (4).

Considering that editors play an important role in the articulation of an ethical framework that influences the conduct of research, the European Association of Science Editors developed a set of guidelines for reporting Sex and Gender Equity in Research (SAGER). These guidelines require authors to report sex and gender information in the title, abstract, study design, data analyses, results, and interpretation of findings (5).

In a review of national research ethics regulations and guidelines in Middle Eastern Arab countries published in 2012, the authors concluded that there is a need for more research on women in the Middle East (6). In the last decade, several Arab and Muslim countries implemented national policies related to the protection of research participants in biomedical research and required investigators to include women as participants.

Fasting is an important ritual in the three major monotheistic religions. Yom Kippour, Carême, and Ramadan are three fasting modalities of abstinence and the opportunity to focus on spirituality. Ramadan fasting (RF) is observed from predawn to sunset for 29–30 consecutive days by two billion Muslims worldwide, mostly living in Asia. The duration of RF varies from 12 to 22 h per day according to the geographical location and solar season. During the month of Ramadan, both men and women refrain from food, water, and sexual activities during daytime. This abstinence does not apply to nighttime.

According to the Holy Quran, travelers, persons with health concerns, elderly children and pregnant, nursing, and menstruating women are exempted from observing RF. Thus, the length of fasting is shorter in premenopausal women (23–25 days) in comparison to men (29–30 days) (7). Since 1952, more than 1,900 articles have been published on the impact of RF on health and disease conditions. Most of these articles (97%) were published in the last three decades (Faris et al., Unpublished data).

Several studies (approximately 250 studies) on healthy participants reviewed recently (8–12) demonstrated that RF induced chronobiological changes (13, 14) and was not associated with adverse metabolic impacts (8, 10–12). Indeed, RF may be accompanied by a moderate improvement of lipid and lipoprotein parameters (15, 16), especially HDL-Cholesterol levels in healthy participants (16), and beneficial effects related to insulin sensitivity, weight and body fat in healthy young men (17). However, RF was associated with a decrease in nocturnal sleep duration (18), daytime alertness, psychomotor performance (13, 19, 20), and rapid eye movement sleep (21) in healthy men. In a healthy population, recent reviews by Faris et al., and Mirmiran et al., also showed that the impact of RF was different in men and women, lipid profile (9, 12, 16), total sleep time (18). Moreover, both HbA1c% and weight decreased only in men with diabetes (22).

The early studies on Ramadan that included men and women were published after 1987 (23) and they included a small number of women. For example, the study done in Malaysia by Husain in 1987 included 12 men and nine women, and the one done by Sajid in 1991 included 46 men and only five women (24).

In patients with type 2, the study of Salti et al., which was conducted in 13 countries including a large sample of patients with diabetes, reported that severe hypoglycemic episodes were significantly more frequent during Ramadan compared with other months in patients with diabetes. Unfortunately, a sex-based analysis was not performed in this study (25). A recent review on diabetes showed that sex was one of the factors that influenced the effect of RF on hypoglycemia events, which were more frequent in women (26). There is currently no reviews addressing the question whether sex has an impact as a biological factor in dtermining the ompacts of RF in patients with diseases such as diabetes, renal, cardiovascular, gastrointestinal diseases, headaches, and epilepsy.

In this study, we conducted a systematic review of all published articles on Ramadan, to determine the effect of sex as a biological factor in disease outcomes of patients who opt to observe RF. The included articles reported data on six diseases: diabetes, renal, cardiovascular, gastrointestinal diseases, headaches, and epilepsy; and reported outcomes such as disease frequencies, physical, clinical, and biochemical parameters.

## Methods

A systematic search of the peer-reviewed published literature was conducted through October 2021, according to the preferred reporting items for systematic reviews and meta-analyses (PRISMA) statement (27), using existing literature in Medline and SCOPUS Electronic databases, with no time limit. The search terms combined Ramadan (or Ramazan or Ramadhan) fasting with one of these pathologies: diabetes, renal diseases, cardiovascular disease (CVD), gastrointestinal diseases (GITDs), headaches, and epilepsy. Reference lists of the obtained studies were hand searched, and authors were contacted to find relevant articles and reviews and to make sure that all related publications were included in the current review.

### Eligibility Criteria

We applied the following inclusion criteria to select the observational and clinical studies: (1) reported data on at least one of these diseases: diabetes, renal, CVD, GITDs, headache; epilepsy; (2) reported data before and during/at the end of Ramadan; (3) compared variables in the same patients' groups; (4) reported descriptive statistics separately for men and women or reporting sex-based statistical comparison; (5) reported on any examination of primary data on disease frequency, complications, physical, biochemical parameters.

We excluded any study meeting at least one of the following exclusion criteria: (1) including only healthy participants; (2) including only one sex; (3) comparing data before and after Ramadan without providing outcomes during the month of Ramadan; (4) comparing only a fasting group to a non-fasting group; (5) including a small population size for men or women (<10 men or <10 women); (6) reporting only data about knowledge, attitude and practice; (7) studies in languages other than English, French, or Arabic.

### Study Screening and Data Extraction

Two authors (RR, DA) removed irrelevant publications such as conference abstracts, reviews, and books, and applied the inclusion and exclusion criteria to the title and abstract. All records that were not excluded based on the title and abstract advanced to the full-text review to confirm eligibility according to the PICOS criteria (Populations, Intervention, Comparisons, Outcomes, and study design), set out in Table 1. Two authors (RR and AN) reviewed the full text of the articles selected from the title and abstract review; reasons for exclusion per article were recorded. The full-text review was done by a first reviewer and then by a second reviewer. The first reviewer made the data extraction, and then the second reviewer verified the results.

**Table 1:** Populations, interventions, comparators, outcomes, and study type (PICOS) criteria.

**PICOS elements**	**Evidence**
**Populations**	Patients with one of these diseases: diabetes, renal, cardiovascular diseases, gastrointestinal diseases, epilepsy, headache, and fasting during Ramadan
**Intervention/exposure**	Ramadan Fasting is the main exposure, and sex is a co-factor
**Comparators**	Comparing male and female changes during Ramadan fasting
**Outcomes**	Any examination of primary data on disease frequency, complications, physical, nutritional and biochemical parameters
**Study type**	Observaitonal/experimental

Using a standardized data-collection form, the following data were extracted from each included study: the first author's last name, publication year, study location, study design, disease condition, population size, number of women, percentage of women, variables, sex effect in baseline, Ramadan effect and sex effect during Ramadan. Data was extracted by RR and confirmed by all authors.

### Outcome Measures

In this study, the main outcome was the comparison between the two sexes of the effect of RF on the outcomes of the disease.

## Results

### Study Characteristics

**Figure 1:**
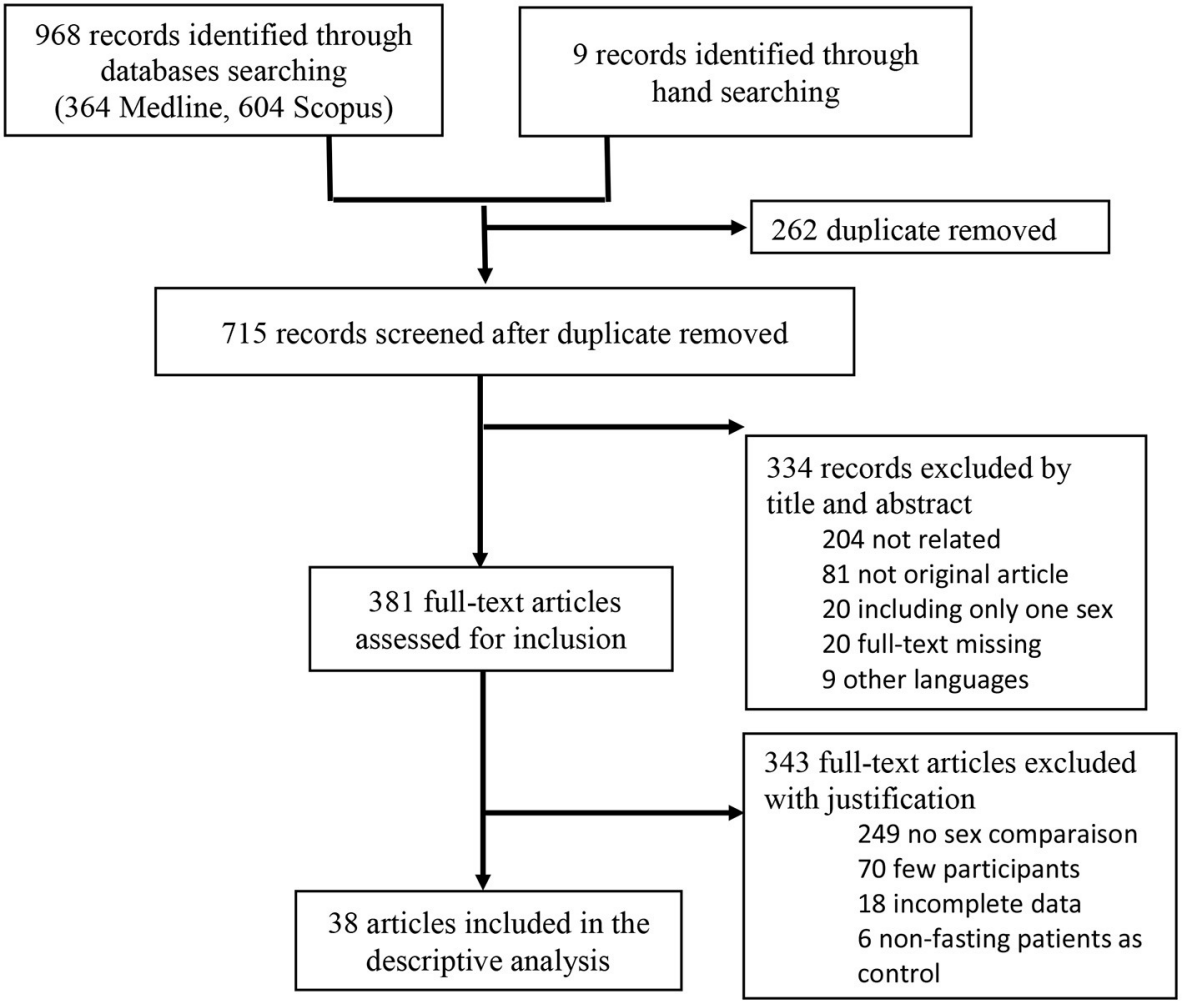
The flow chart with the different phases of the systematic review according to the PRISMA statement.

In this review, the primary search, after removing the duplicated, identified 715 citations from databases and the bibliography of the full-text articles. After the first screening phase of titles and abstracts, we excluded 334 articles because they did not meet the eligibility criteria especially reporting descriptive statistics related to sex-based differences in patients fasting during Ramadan. A total number of 381 articles appeared to be relevant for the full-text analysis. The second screening phase of the full-text articles resulted in the exclusion of 343 additional articles, and 38 articles remained for data extraction. The search strategy is presented in Figure 1. Of the included studies, 19 were retrospective, 12 were prospective studies, and six were cross-section surveys, and only one study was a randomized clinical trial.

Publications reporting sex-related statistics during Ramadan represented only 10.0% of the total publications on RF and diseases including men and women (Table 2). This percentage was lower (4%) for the studies on Ramadan and diabetes.

**Table 2:** Number and percentage of the studies reporting data for both men and women or reporting comparison between the two sexes during Ramadan fasting in the patients with diabetes, renal, cardiovascular, gastrointestinal diseases, headache, and epilepsy.

**Disease**	**Number of original articles including men and women**	**Number of articles reporting sex comparison**	**% of articles reporting sex comparison**
Diabetes	248	10	4.0%
Renal	52	7	13.5%
Cardiovascular	52	8	15.4%
Gastrointestinal	25	8	32.0%
Headache	10	6	60.0%
Epilepsy	8	4	50.0%
**Total**	**381**	**38**	**10.0%**

The total number of the patients was 25,023 of which 44.4% were female, and the mean age was 47.7 years. These studies reported general data from Ramadan and baseline time-points, i.e., before Ramadan. The sex effect was not always statistically analyzed. Only seven articles reported the odds ratio or relative risk for sex effect on observers of Ramadan, 18 articles reported a *p*-value for the comparison between men and women during Ramadan, eight articles reported the effect of RF in both sex separately, and five articles did not report a *p*-value.

The articles included in this review reported data for diabetes, renal diseases, GITDs, CVD, headaches, and epilepsy; and were conducted in 18 countries, mostly from Turkey (nine articles), Iran (five articles), Saudi Arabia (four articles), Egypt and Qatar (three articles), Morocco (two articles), Bahrain, Iraq, Israel, Jordan, Kuwait, Lebanon, Libya, Malaysia, Mali, Pakistan, Singapore, Tunisia (one article each). Most of these articles were published in the last 20 years, and were conducted in places where Ramadan fell in summer (27 articles, 71%) or autumn (10 articles, 26%); only one study was conducted in spring.

### Sex Factor in Diabetes Diseases During Ramadan

**Figure 2:**
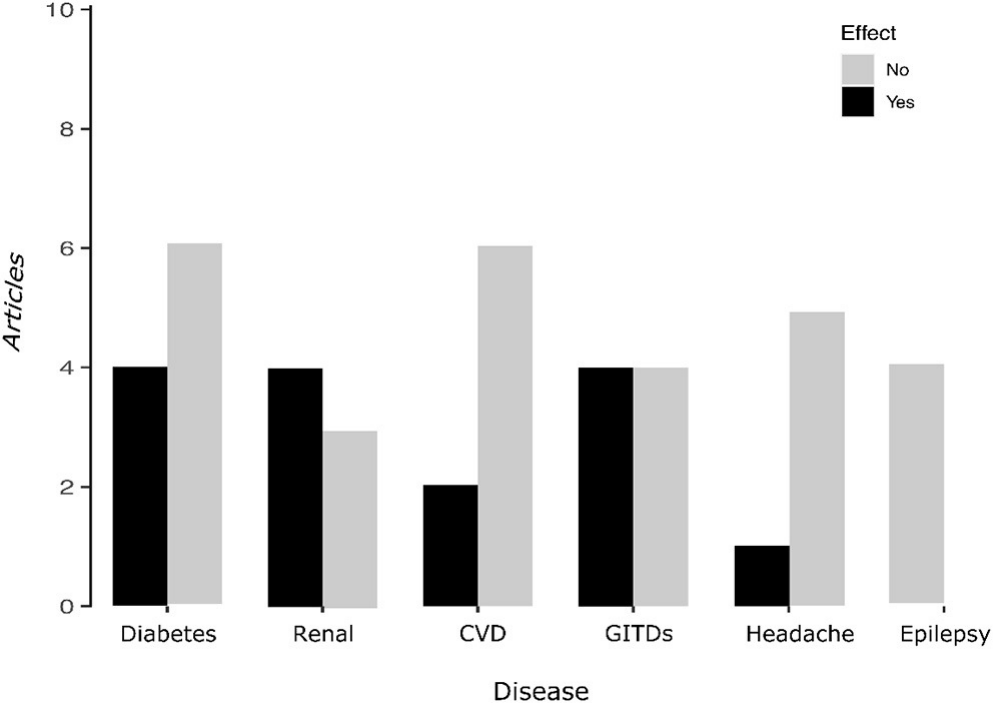
The Bar-plot summarizes the results obtained from studies analyzed and shows the articles that reported sex-related changes during Ramadan. 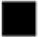 Number of articles reporting differences between men and women during Ramadan fasting 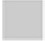 Number of articles reporting no differences.

A total of 10 observational studies including 3,936 patients, of which 46.3% women, with diabetes diseases, have reported data (28–37) for fasting men and women with type 2 diabetes or Type 1 Diabetes (Table 3). A large number of variables were reported in these studies, but we focused in this review on body mass index (BMI), blood glucose (BG), HbA1c, insulin resistance (IR), hypoglycaemia, hyperglycaemia, diabetic ketoacidosis, and emergency admission. No sex effect was reported in the baseline conditions before Ramadan except for BMI, which was higher in women (31, 32) (Figure 2).

**Table 3:** Sex differences in outcomes related to diabetes in patients before and during Ramadan fasting.

**Studies (*n* = 10)**	**Country**	**Study design**	**Participants**	**Sample size**	**No. of women**	**% of women**	**Age (year)**	**Variables**	**Sex effect in baseline**	**Ramadan effect**	**Sex effect during Ramadan**
Yarahmadi e al. (28)	Iran	PS	T2D	57			Adults	BG. IR	Not given	No effect	BMI increased in W and decreased in M. IR decreased in M. No effect in BG
M'guil et al. (29)	Morocco	PS	T2D	120	62	51.7%	Adults	BG. HbA1c. BMI	Not given	Glucose decreased. No effect for HbA1c. BW. BMI	Glucose decreased in W. No effect for HbA1c. BMI
Elmehdawi et al. (30)	Libya	RS	T2D. T1D	270	142	52.6%	Adults	Frequency of DKA	Not given	DKA decreased. DKA was more frequent in T1D than in T2D	No sex effect
Traoré et al. (31)	Mali	PS	T2D	24	11	45.8%	48.9	BW	BW higher in W	Not given	BW decreased in M
Bener and Yousafzai (32)	Quatar	PS	T2D. T1D	1,301	626	48.1%	45.9	BMI. BG. HbA1c.	BMI higher in W	BG and HbA1c decreased	No sex effect
Yeoh et al. (33)	Singapore	PS	T2D	29	14	48.3%	57.5	BMI. HbA1c	No sex effect	Decreased	No sex effect
AlKhaldi et al. (34)	Saudi Arabia	CSS	T1D. T2D	378	186	49.2%	45.5	Frequency of HypoG	Not given	Increased	Increased in W
AlAssaad et al. (35)	Lebanon	RS	Diabetes	553	220	39.8%	54.1	Frequency of admission	No effect	No effect	No effect
Abid eet al. (36)	Tunisia	CSS	T1D. T2D	526	220	41.8%		HypoG. fasting frequency		Severe HypoG increased in T2D	No sex effect in hypoG. More men fasted
Zaghlol et al. (37)	Jordan	RCT	T2D	678	342	50.4%	58	HypoG. HyperG	Not given	Not given	No sex effect in HypoG. HyperG
**Total and mean**				**3,936**	**1,823**	**46.3%**	**57.8**				

*CSS, cross-sectional survey; PS, prospective study; RS, retrospective study; RCT, randomized clinical trial; BG, blood glucose; IR, insulin resistance; HbA1c, glycated hemoglobin A1c; BMI, body mass index; DKA, diabetic ketoacidosis, BW, body weight; HypoG, hypoglycemia; HyperG, hyperglycemia; M, men; W, women*.

During Ramadan, a decrease in blood glucose BG (29, 32), HbA1c (32, 33), BMI (33), and diabetic ketoacidosis (30) was reported in four studies; while severe hypoglycaemia was reported to be increased in women (34, 36). RF effect was not significant for BG (28), Insulin resistance (28), HbA1c (29), BMI (29), and the frequency of emergency admission (35).

Sex effect during Ramadan was reported to be not significant in seven studies for BMI (32, 33), BG (28, 29, 32, 33), HbA1c (29, 32, 33), Hypoglycaemia (36, 37) the frequency of emergency admission (35). However, the sex effect during Ramadan was reported to be significant in four studies for BG (29), BMI (28, 31), IR (28), and hypoglycaemia (34). Thus, during Ramadan, BMI and body weight decreased in men and increase in women (28, 31), BG decreased and hypoglycaemia increased in women only (29, 34), and insulin resistance decreased in men only (28).

### Sex Factor in Renal Diseases During Ramadan

A total of seven studies including 4,193 patients of which 36.2% were women have reported sex-related effects in renal diseases (35, 38–43), especially in patients with the diagnosis of renal colic disease, chronic kidney disease (CKD), hemodialysis, and transplant recipients (Table 4). In the period before Ramadan, most of these studies showed sex-related effects in the frequency of renal diseases, with renal colic more frequent in men (35, 39, 41, 43). During Ramadan, hospital admissions for renal colic disease were higher in three studies (35, 38, 43) and mortality was higher in one study (40). The sex effect during Ramadan was significant in three studies which reported an increase in renal colic frequency in men only (35, 39, 43), and an increase in mortality in female hemodialysis patients (40). In patients admitted for CKD, the glomerular filtration rate increased during Ramadan but did not show sex effects during this month (38). Biochemical parameters did not show RF or sex effect (42).

**Table 4:** Sex differences in outcomes related to renal diseases in patients during and outside the month of Ramadan.

**Studies (*n* = 7)**	**Country**	**Study design**	**Participants**	**Sample size**	**No. of women**	**% of women**	**Age (year)**	**Variables**	**Sex effect in baseline**	**Ramadan effect**	**Sex effect during Ramadan**
Basiri et al. (39)	Iran	RS	Renal colic	574	176	30.7%	36.4	Frequency	Higher in M	No effect	Increased in men
NasrAllah and Osman (38)	Egypt	CSS	CKD	131	72	54.9%	59.7	Creatinine	Not given	Increased	No sex effect
Imtiaz et al. (40)	Pakistan	RS	hemodialysis	1,841	785	42.6%	60	Mortality	Not given	Increased	Increased in W
Al Assaad et al. (35)	Lebanon	RS	Renal colic	514	148	28.8%	54.1	Frequency	Higher in M	Increased	Increased in men
Al Mahayni et al. (41)	Saudi Arabia	CSS	Renal colic	237	59	24.9%	45.8	Frequency	Higher in M	No effect	No sex effect
Adanan et al. (42)	Malaysia	PS	Hemodialysis	87	39	44.8%	54.3	Physical, Nutritional parameters	Not given	Nondetrimental changes	No sex effect
Mustafa et al. (43)	Bahrain	RS	Renal colic	809	237	29.3%	40	Frequency	Higher in M	Increased	Increased in men
**Total and mean**				**4,193**	**1,516**	**36.2%**	**50.0**				

*RS, retrospective study; PS, prospective study; CSS, cross-sectional survey; GFR, Glomerular Filtration Rate; CKD, Chronic Kidney disease; M, men; W, women*.

### Sex Factor in Cardiovascular Diseases During Ramadan

A total of eight observational studies including 6,111 patients, of which 42.9% were women, with CVD (Table 5) have reported data for fasting men and women (35, 44–50). In baseline conditions, the frequency of congestive heart failure (CHF), stroke, and the stroke scale was higher in men (35, 45, 50), while cerebral vein thrombosis (CVT) was higher in women (48, 49). During Ramadan, several studies showed no significant effect of fasting on the frequency of stroke (35, 44), congestive heart failure (45), Acute Myocardial Infarction (47); and biochemical parameters in stable patients with heart diseases (46). However, four studies reported a significant increase in CVT incidence, stroke score, and major adverse cardiac events (MACEs) (48–50).

**Table 5:** Sex differences in outcomes related to cardiovascular diseases in patients during and outside the month of Ramadan.

**Studies (*n* = 8)**	**Country**	**Study design**	**Participants**	**Sample size**	**No. of women**	**% of women**	**Age (year)**	**Variables**	**Sex effect in baseline**	**Ramadan effect**	**Sex effect during Ramadan**
Akhan et al. (44)	Turkey	RS	Stroke	1,579	797	50.5%	65	Frequency	No effect	No effect	No sex effect
Al Suwaidi ET AL. (45)	Qatar	RS	CHF	2,160	870	40.3%	64.2	Frequency	higher in M	No effect	No sex effect
Chamsi-Pasha and Ahmed (46)	Saudi Arabia	PS	Heart diseases stable patients	86	32	37.2%	56.3	Clin. biochem parameters	CV	No effect	No sex effect
Topacoglu et al. (47)	Turkey	RS	AMI	817	238	29.1%	59	AMI	Not given	No effect	No sex effect
Saadatnia et al. (48)	Iran	RS	CVT	162	133	82.0%	35.2	CVT incidence	Higher in W	Increased	Increased incidence of CVT in W taking OCP
Sasannejad et al. (49)	Iran	RS	CVT	70	59	84.0%	35	CVT incidence	Higher in W	Increased	Increased incidence of CVT in W taking OCP
Salama and Belal (50)	Egypt	PS	Cerebral stroke	1,062	420	40.0%	63.5	NIHSS	Higher in M	increased	No sex effect
AL Assaad et al. (35)	Lebanon	RS	Stroke	175	73	41.7%	54.1	Frequency	Higher in M	No effect	No sex effect
**Total and mean**			**6,111**	**2,622**	**42.9%**	**53.0**				

*RS, retrospective study; PS, prospective study; CSS, cross-sectional survey; CHF, congestive heart failure; CVT, cerebral vein thrombosis; AMI, acute myocardial infarction; NIHSS, National Institutes of Health Stroke Scale; MACEs, major adverse cardiac events; DAS, disease activity score; OCP, oral contraceptive pills; M, men; W, women*.

No sex effect was reported during Ramadan for the incidence of stroke, CHF, Acute Myocardial Infarction, or MACEs, except the incidence of CVT which was reported to be significantly higher for fasting women, especially in women taking oral contraceptive pills (48, 49).

### Sex Factor in Gastrointestinal Diseases During Ramadan

A total of eight papers with 5,409 patients of which 32.5% were women have reported statistical comparison between men and women on the frequency of GITDs (51–57). In the baseline period, sex effect was significant in most of these studies, since gastrointestinal diseases [Acute Upper Gastrointestinal Bleeding (AUGIB), Peptic Ulcer Perforation PUP, Appendicitis] were reported to be more frequent in men than women (51–58); except for acute cholecystitis disease which was higher in women (53).

During Ramadan the frequency of admission for AUGIB and PUP increased (51, 52, 54, 55, 57, 58). The sex effect during Ramadan was reported to be not significant in most of the included studies (53–56, 58). However, the study of Dönderici et al. (51), showed that female patients tended to develop more hemorrhage and perforations during Ramadan than male patients; and that of Gokakin et al. demonstrated that PUP increased in women only during Ramadan (55). On the others hand, surgery (52) and admission (57) for PUP increased in men only (Table 6).

**Table 6:** Sex differences in outcomes related to gastrointestinal diseases in patients during and outside the month of Ramadan.

**Studies (*n* = 8)**	**Country**	**Study design**	**Participants**	**Sample size**	**No. of women**	**% of women**	**Age (year)**	**Variables**	**Sex effect in baseline**	**Ramadan effect**	**Sex effect during Ramadan**
Dönderici et al. (51)	Turkey	RS	AUGIB. PUP	1,114	862	77.4%	45.5	Frequency	Higher in M	Increased	Increased in W
Kucuk et al. (52)	Turkey	RS	surgery for PUP	260	8	3.1%	40.5	Frequency	Higher in M	Increased	Increased in M
Hosseini et al. (53)	Iran	CSS	AC	141	87	61.7%	56.3	Frequency	Higher in W	No effect	No effect
Ozkan et al. (54)	Turkey	PS	AUGIB	71	21	29.6%	57.2	Frequency	Higher in M	Increased	No effect
Sulu et al. (56)	Turkey	RS	AA	992	390	39.3%	28.8	Surgery	Higher in M	No effect	No effect
Gokakin et al. (55)	Turkey	RS	PUP	229	34	14.9%	45	Surgery	Higher in M	Increased	Increased in W and decrease in men
Elmekkaoui (58)	Morocco	RS	AUGIB	291	108	37.1%	49.7	Frequency	Higher in M	Increased	No effect
Kocakusak et al. (57)	Turkey	RS	PUP	2,311	249	10.8%	40	Frequency	Higher in M	Increased	Increased in men
**Total and mean**				**5,409**	**1,759**	**32.5%**	**42.5**				

*RS, retrospective study; CSS, cross-sectional survey; PS, prospective study; AUGIB, acute upper gastrointestinal bleeding; PUP, peptic ulcer perforation; AC, acute cholecystitis; GIT, gastro-intestinal tract; AA, acute appendicitis; M, men; W, women*.

### Sex Factor in Headache During Ramadan

A total of six studies with 4,688 patients of which 65.2% were women have reported statistical comparison between men and women on headache. Most of these studies reported that in the baseline condition, the headache was more frequent in women (35, 47, 59–61) and that RF was a triggering factor for headache (47, 59–61). The sex effect during Ramadan was reported to be not significant in most of these studies (35, 47, 59–61); except for one study realized in Qatar which reported an increase in headache in men during Ramadan (Table 7). Only one study showed that headaches increase during Ramadan in men only (62).

**Table 7:** Sex differences in the frequencies of Headache in patients during and outside the month of Ramadan.

**Studies (*n* = 6)**	**Country**	**Study design**	**Participants**	**Sample size**	**No. of women**	**% of women**	**Age (year)**	**Variables**	**Sex effect in baseline**	**Ramadan effect**	**Sex effect during Ramadan**
Topacoglu et al. (47)	Turkey	RS	Uncomplicated headache	2,582	1,841	71.3%	42.2	Frequency	Higher in W	Increase	No effect
Bener et al. (62)	Qatar	CSS	Clinics attenders	688	337	48.9%		Frequency	Not given	Not given	Higher in M
Abu-Salameh et al. (59)	Israel	RS	Migraine	32	23	71.9%	34.4	Frequency	Higher in W	Increase	No effect
Al-Shimmery et al. (60)	Iraq	RS	Migraine	200	154	77.0%		Frequency	Higher in W	Increase	No effect
Al Assaad et al. (35)	Lebanon	RS	Headache	893	443	49.6%	54.1	Frequency	Higher in W	No effect	No effect
Al-Hashel et al. (61)	Kuwait	RS	Migraine	293	260	88.7%	37.1	Frequency	Higher in W	Increase	No effect
**Total and mean**				**4,688**	**3,058**	**65.2%**	**41,95**				

*RS, retrospective study; M, men; W, women*.

### Sex Factor in Epilepsy Frequency During Ramadan

Only four studies including 686 patients of which 47.7% were women have reported statistical comparison between men and women on epilepsy (35, 63–65). The comparison between men and women was not given in three studies epilepsy (35, 63–65), and only one study showed that the frequency of epilepsy was not different in men and women in the period outside Ramadan (35). During Ramadan, the four studies reported no significant differences between men and women in the risk of developing epileptic seizures or seizures frequencies during RF (Table 8).

**Table 8:** Sex differences in the frequencies of epilepsy in patients during and outside the month of Ramadan.

**Studies (*n* = 4)**	**Country**	**Study design**	**Participants**	**Sample size**	**No. of women**	**% of women**	**Age (year)**	**Variables**	**Sex effect in baseline**	**Ramadan effect**	**Sex effect during Ramadan**
Gomceli (64)	Turkey	PS	EpilePSy	114	62	54.4%	31.6	Frequency	Not given	Increase	No effect
Al Assaad et al. (35)	Lebanon	RS	EpilePSy	105	39	37.1%	54.1	Frequency	No effect	No effect	No effect
Alqadi et al. (63)	Saudi Arabia	PS	EpilePSy	37	15	40.5%	30.0	Frequency	Not given	decrease	No effect
Magdy et al. (65)	Egypt	PS	EpilePSy	430	211	49.1		Frequency	Not given	No effect	No effect
**Total and mean**			**686**	**327**	**47.7%**	**30.0**				

*PS, prospective study; RS, retrospective study; M, men; W, women*.

## Discussion

This systematic review included 38 studies reporting sex-based outcomes during Ramadan month, with primary data from 25,023 patients fasting and with one of these pathologies: diabetes, renal diseases, GITDs, CVD, headaches, and epilepsy, and of whom 44.4% were women. Of the total number of studies (*n* = 381), only 38 studies (10.0%) reported results in men and women separately or reported sex-based comparison during RF. This percentage was lower for the studies on Ramadan and diabetes (4%) (Figure 2).

Sex-based differences during Ramadan was reported by 18 studies for several variables such as BMI (28, 31), blood glucose (36) frequency of hypoglycemia (34), frequency of fasting (36), in patients with diabetes; renal colic (35, 39, 40, 43), mortality, thrombosis (48, 49), AUGIB and peptic ulcer perforation (51, 52, 57, 66). However, 21 studies reported that the sex factor was not associated with the effect of RF in diabetes (28–30, 32), renal diseases (38, 41, 42), CVD (44–46, 50, 67) GITDs (53–58), headache and epilepsy (59–61, 63–65, 67). Most of these differences between men and women were not specific to RF because they were also reported in the baseline period. Indeed, during the period outside Ramadan, the frequency of renal colic, CHF, stroke, GITDs diseases were higher in men; while BMI, CVT, and headache were higher in women.

Some methodological aspects must be considered when interpreting the results of these studies. First, we included studies that showed results in men and women separately, even though the sex effect was not analyzed directly for observers of RF. Only 25 studies reported direct sex-based comparison during Ramadan.

Second, we could not give a quantitative summary of evidence substantiating the association between the fasting effects and the sex factor, as there were limited available quantitative data based on sex comparison. Additionally, most of the included studies did not mention “sex” or “gender” as a factor in the title or the objective of the study. Only one article specified sex differences in the title (33) but the sex aspect was not mentioned in the introduction and as one of the study objectives. In one study, only “women” was mentioned in the title even though both men and women were included in the study (49). Finally, we excluded the studies that compared fasting patients with non-fasting patients during given days in Ramadan, because, in this protocol, patients in the two groups did not have the same characteristics in the period before Ramadan. For example, in four studies (68–71) the fasting group and the non-fasting group did not have the same age; and in one study they did not have the same comorbidity status (72) which could bias the RF effects. We also excluded the studies comparing data before and after Ramadan without providing outcomes during the month of Ramadan (22, 73) because changes recorded several weeks after Ramadan could be different than changes assessed during Ramadan.

Additional methodological challenging issue during Ramadan is related to the disparity in the number of fasting days for men and women. According to Muslim rules, menstruating women cannot fast during Ramadan and up to 40 days following childbirth. Also, women are allowed not to fast during pregnancy and lactation. This implies that women have fewer fasting days in comparison to men. The length of fasting is 23–25 days in women and 29–30 days in men (7). This disparity could represent a challenge for researchers since they have to take into consideration the number of fasting days during Ramadan to compare disease outcomes. Despite this challenge, sex-based research in Ramadan is needed.

Regarding the sex factor in diabetes outcome, several large studies and systematics reviews showed that many aspects of energy balance and glucose metabolism are regulated differently in males and females and influence their predisposition to type 2 diabetes (74). In the non-diabetic population, men had higher fasting plasma glucose and HbA1c levels than women (75). In patients with Type 2 diabetes, women had greater reductions in BG, and higher annual rates of severe hypoglycemia than men (76). Moreover, in a study using data from 751 studies including 4,372,000 adults from 146 countries, diabetes was reported to be more prevalent in men than in women, with higher prevalence in the Middle East and North Africa in comparison to western countries (77). In a recent review about metabolic health in the Middle East and North Africa, it was reported that Middle Eastern and North African women have the highest risk of metabolic diseases compared to women globally (78).

RF would be acceptable for patients with well-balanced diabetes who are conscious of their disease and compliant with their diet and drug intake, especially in patients who had received focused individualized diabetic education sessions and antidiabetic medications adjustment before and after Ramadan (9, 26, 79, 80). However, an increase in hypoglycemia attacks during Ramadan was reported (25, 34, 36).

During Ramadan, BMI and body weight decreased in men and increased in women, BG decreased and hypoglycaemia increased in women only. Differences between men and women in knowledge, attitude, and practice may explain part of these results during Ramadan. It has been shown that more women than men broke the fast in relation to diabetes concerns (81, 82). Additionally, women changed their medications during Ramadan (81) and have lower drug adherence than men, especially for high-cost drugs (83).

As in diabetes, kidney dysfunction was also shown to be influenced by the sex factor, and the female sex is shown to be reno-protective (84–86). Estrogen hormones could play a key role in this protective effect. A recent review reported that experimental and clinical studies have shown that targeting estrogen signaling pathways might have protective effects against certain renal disorders (87). Despite this protection of females against the initiation of kidney disease, the progression of multiple nephropathies displays the worst outcomes amongst female patients, in comparison to men (88). A large study that assessed dialysis outcomes in the Human Mortality Database with 206,374 patients receiving hemodialysis from 12 countries have demonstrated that fewer women than men were undergoing hemodialysis treatment and that the survival advantage that women have over men in the general population was markedly diminished in hemodialysis patients (89). In the present review, most of the studies also reported that renal diseases were more frequent in men before and during Ramadan. Two studies (40, 42) reported that fewer women (42.6 and 44.8%) received hemodialysis in the baseline period. The study by Imtiaz et al. (40) reported that the mortality rate increased in hemodialysis patients during Ramadan (10.5% during Ramadan vs. 6.9%−9.5% outside Ramadan), with more female patients undergoing hemodialysis that die (53%) during Ramadan. But, this study did not report a direct sex-based comparison of mortality during Ramadan. Additionally, data from different countries reported that the likelihood of women being registered on transplant waiting lists is less than men (90); and therefore, they are less likely to receive organ transplants. Still, the relationship between mortality in hemodialysis female patients and health service access needs to be more studied.

The prevalence of CVD was known to be higher in men (91). Recently, the sex-based differences in stroke have been questioned, as some controversial results were obtained in clinical and population studies. It was reported that women are more affected by stroke, exhibiting higher mortality and disability rates post-stroke than men. Also, the incidence of strokes rises in young women (92). In this review, the incidence and frequency of CVD did not change during Ramadan, except for CVT incidence, which increased. Jahrami et al. (12), reported in a recent meta-analysis that RF may confer short-term transient protection against CVD among healthy people. Another review concluded that studies on RF in patients with cardiac disease are sparse, observational, of small sample size, and have short follow-ups (93). No sex effect was reported during Ramadan for the incidence of stroke, CHF, Acute Myocardial Infarction, or MACEs. However, the incidence of CVT was higher for fasting women taking oral contraceptive pills. According to Ghiasian Jahrami et al. (94), women in Moslem countries use OCPs to delay their menstruation and postpone their period during Ramadan, which will allow them to observe the fasting during the entire month. This last study showed also that fasting in patients with CVT using OCPs causes a significant increase in the focal neurological deficit and hemorrhage (94). Dehydration during Ramadan dehydration was the most common risk factor for CVT among fasting women (95).

As in diabetes, renal, and CVD, sex-based differences in GITDs, such as gastro-esophageal reflux and peptic ulcer diseases are reported by several large studies and were more frequent in men (96, 97). It is important to mention that these differences were found mainly among young men and young women. In postmenopausal women, the prevalence of GITDs increases more rapidly in women than men so the prevalence in elderly patients is similar in both sexes or greater in women (97, 98). In the same way, Ye et al. (99) demonstrated that the integrity of the esophageal mucosa is more fragile in males than in females; and that the esophageal mucosal barrier attenuates more rapidly with increasing age in females than in males. These findings suggest that exposure to estrogen during the reproductive years in women protects them from the onset of several GITDs (98).

In this review, in the baseline condition, and as it was described in the literature, AUGIB and PUP were more frequent in men than women, except for acute cholecystitis which was higher in women. During Ramadan, GITDs increased. The study of Iraki et al. (100) suggested that the modifications of feeding and sleeping schedule during Ramadan induced a decrease in gastric pH, and could be a risk factor for the duodenal ulcer. The results related to the differences between men and women during Ramadan were controversial. The study of Dönderici et al., and that of Gokakin et al. showed that the AUGIB and the PUP increased more in fasting women while the finding of Kucuk and Kocakusak showed an increase in fasting men for the PUP. In these two studies, the mean age of the population was younger than in the studies by Dönderici et al., and Gokakin. Age was demonstrated to be an important factor in sex-based differences in several diseases. For this reason, it is important to take into consideration the age factor while comparing outcomes in men and women during Ramadan.

Regarding Headache, in the period outside Ramadan, the headache was more frequent in women. In a recent large study, migraine and severe headaches were considered by authors serious public health issues, In 2018, the prevalence was 15.9% across all adults, with women more affected than men (21% of women and 10.7% of men) (101). In a recent review, migraine over the life course occurs in women three to four times more often than in men (102). As in diabetes and renal diseases, estrogen fluctuation was thought to be involved in the explanation of the disparity in migraine between men and women (102).

During Ramadan, headaches and migraine increased in both men and women. The mechanisms triggering or worsening headaches during fasting are still hypothetical. One of the hypotheses is related to the change in the circadian rhythms of food intake and the oxidative state. Both fasting and eating a large meal could disturb the homeostatic state. Several studies reported that meal skipping might be one of the major causes of headaches and that minimizing daily blood glucose fluctuations might help prevent migraines and headaches (103–105). In the review of Torelli Jahrami et al., hypoglycemia and caffeine withdrawal have been especially implicated as causative factors in headache during fasting. The study of Bener et al. reported that the frequency of headaches increased only in men (62). The coffee and tobacco privation during Ramadan daytime may explain part of these specific increases in headache in fasting men since smoking is more frequent in men than in women in Moslem countries (106, 107).

During Ramadan, noting that little progress has been made in the reporting differences between men and women with diseases, it is hazardous to make any conclusion about the sex-related differences during fasting. It is important to emphasize that any sex based changes in health outcomes during RF may be confounded by other factors such as lifestyle, knowledge, attitude, practice, health access, etc.

## Conclusion

Currently, small attention is paid to sex as a determinant factor in patients while fasting during Ramadan. There appeared to be differences in the frequency and incidence of diseases in men and women during Ramadan. The results should be interpreted with caution as only a few studies reported results for men and women separately. The authors suggest that closer attention to sex differences regarding the frequency and the progression of the diseases may help to improve the quality of the study and the patient care, especially to benefit those patients willing to fast during Ramadan.

## Data Availability Statement

The original contributions presented in the study are included in the article/supplementary material, further inquiries can be directed to the corresponding author.

## Author Contributions

RR and MF jointly developed the research questions and the research methods for the review. RR, NA, and DA selected studies from the title and abstract and reviewed the full text of the relevant articles. RR and NA made the data extraction. RR was a major contributor to writing the manuscript and MF provides edits. All authors contributed to the final versions of the manuscript, read and approved the final manuscript.

## Conflict of Interest

The authors declare that the research was conducted in the absence of any commercial or financial relationships that could be construed as a potential conflict of interest.

## Publisher's Note

All claims expressed in this article are solely those of the authors and do not necessarily represent those of their affiliated organizations, or those of the publisher, the editors and the reviewers. Any product that may be evaluated in this article, or claim that may be made by its manufacturer, is not guaranteed or endorsed by the publisher.

## Abbreviations

AUGIB: acute upper gastrointestinal bleeding
BG: blood glucose
BMI: body mass index
CHF: congestive heart failure
CKD: chronic kidney disease
CVD: cardiovascular diseases
CVT: cerebral vein thrombosis
GITDs: gastro-intestinal tract diseases
HbA1c: glycated hemoglobin A1c
MACEs: major adverse cardiac events
PUP: peptic ulcer perforation
RF: Ramadan fasting.
